# Knowledge and beliefs about oocyte cryopreservation for medical and social reasons in female students: a cross-sectional survey

**DOI:** 10.1186/s12905-023-02481-2

**Published:** 2023-06-24

**Authors:** Mohammad Mehdi Akhondi, Zohreh Behjati Ardakani, J. Catja Warmelink, Shima Haghani, Fahimeh Ranjbar

**Affiliations:** 1 Reproductive Biotechnology Research Center, Avicenna Research Institute, ACECR, Tehran, Iran; 2 University of Groningen, University Medical Center Groningen, Department of Primary and Long-term Care, University of Groningen, Groningen, the Netherlands; 3 Midwifery Academy Amsterdam Groningen, InHolland, Groningen, the Netherlands; 4 Amsterdam UMC location Vrije Universiteit Amsterdam, Midwifery Science, Amsterdam, the Netherlands; 5 Nursing and Midwifery Care Research Center, Health Management Research Institute, Iran University of Medical Sciences, Tehran, Iran

**Keywords:** Oocyte cryopreservation, Fertility preservation, Social egg freezing, Fertility sparing treatment, Age-related infertility

## Abstract

**Background:**

With the increasing number of young women surviving cancer and a growing trend among highly educated women to postpone childbearing for educational or professional pursuits, there is a rising demand for egg freezing services to ensure a successful pregnancy. This study aims to assess the knowledge and beliefs surrounding oocyte cryopreservation, both for medical and social reasons, among female students in Tehran, Iran.

**Methods:**

An online cross-sectional survey was carried out from March to August of 2022, involving a total of 1279 childless students pursuing master’s and doctoral degrees at universities in Tehran. The participants were between the ages of 18 and 38. Knowledge and beliefs about medical and social oocyte cryopreservation were assessed through Fertility Preservation Survey (FPS) instrument.

**Results:**

The mean age of the participants was 26.38 ± 4.9. The majority of students expected to be “30–34 years” when they become pregnant with their first child (41.1%, M: 30.3 ± 4.13 years) and “35–39 years” when they give birth to their last child (46.7%, M: 35.28 ± 4.18 years). The students agreed with preserving fertility with medical (93.3%) and social (86.9%) indications and believed the medical (95.1%) and social (87.4%) costs of cryopreservation should be covered by the healthcare system. Among the participants, 75.6% considered cost to be a definite or probable factor in their decision to pursue fertility preservation. The oncology team’s recommendation was identified as the most important factor in deciding on medical egg freezing (92.6%, M: 3.46 ± 0.71). The overall correct response rate for the knowledge questions was 57.7%. The majority of participants (95.5%) agreed that physicians should routinely provide information about egg freezing to women of childbearing age during their regular healthcare visits.

**Conclusions:**

The research results revealed that female students in Tehran universities have a positive attitude towards medical and social egg freezing, but lack sufficient knowledge about the ideal timing of childbearing. Health professionals could provide detailed information about fertility preservation and age-related infertility as part of routine healthcare visits or reproductive health planning. Additionally, expanding supportive policies and incentives for childbearing established by the government to cover the costs of fertility preservation would be beneficial.

## Background

Recent studies suggest that ovarian aging, whether physiological or pathological, limits female reproductive capacity [1], and oocyte cryopreservation (OC) is an evolving branch of reproductive medicine that may be increasingly used by women to ensure their ability to conceive [2, 3]. As a woman ages, her ovarian reserve, as well as the quantity and quality of her eggs, decreases, with conception before the age of 35 being the safest way for a woman to have children. By freezing and storing eggs, women can maintain their reproductive ability at an older age, which has been proposed as a form of “fertility insurance” against age-related infertility[4, 5]. In recent years, cancer treatment advances have significantly increased cancer patient survival rates [6], and medical OC provides women with the opportunity to preserve their eggs for future conception [2]. However, few women diagnosed with cancer choose fertility preservation (FP) strategies, mainly due to a lack of knowledge [7].

Around 50% of women with endometriosis experience infertility and are susceptible to a decreased ovarian reserve due to the disease’s pathophysiological mechanisms. While the approach to treating endometriosis patients remains controversial, utilizing a multidisciplinary approach that includes surgical procedures, assisted reproductive technologies, and FP technologies is crucial for achieving positive outcomes. Women with endometriosis who are at risk of bilateral ovarian injury, such as those with bilateral endometriomas, may consider FP through oocyte or ovarian tissue cryopreservation on an individual basis [8–10]. Egg freezing is also an alternative for women with a family history of early menopause and for individuals who cannot freeze embryos due to religious or ethical beliefs. Women who delay marriage and childbearing for reasons such as education, employment, difficulty finding a suitable partner, financial instability or personal reasons [11, 12] can also benefit from non-medical or social egg freezing, which allows them to preserve their fertility [2, 13, 14].

It is still too early to draw final conclusions about the outcomes of medical and social OC but initial data suggests its utilization. However, egg freezing can cause new ethical and financial problems, and childbearing at an advanced maternal age can pose risks to both the mother and the child [15]. In a recent study, the pregnancy rate per transfer and per patient were reported as 31% and 41%, respectively [15]. While egg freezing can prevent age-related infertility, it may also result in new ethical and financial concerns, as well as risks for both the mother and child if childbirth occurs at an advanced maternal age (≥ 40 years) [16]. Additionally, women who undergo this procedure may remain fertile or ultimately choose not to pursue parenthood [17]. However, the low awareness but positive attitude about FP was reported in recent studies [11, 17–19], suggesting a growing demand for egg freezing services [20].

The fertility rates in Iran have significantly decreased in the last thirty years [21]. This decrease can be attributed not only to economic problems but also to delayed marriage and childbearing as well as infertility issues that show significance of raising awareness about FP options [22]. Despite having one of the strongest ART industries in the world, there is a lack of sufficient data and limited studies in this field in Iran and other Islamic countries in the Middle East region. Studies conducted in Iran have revealed a significant knowledge gap among candidates for social egg freezing [23], adult cancer patients and their parents [24], as well as oncologists [25], regarding age-related fertility decline, FP options, and its associated complications. To our knowledge, there is also no available data on the acceptability of medical and social indications of FP among female Iranian students. With the increasing number of young women surviving cancer and highly educated women postponing childbearing for professional pursuits [1, 26], this study aims to evaluate the knowledge and beliefs about OC for both medical and social reasons among female students in Iran.

## Methods

### Study design

This cross-sectional study investigated female students’ knowledge and beliefs about medical and social OC in Tehran universities. Tehran, with a population of 14 million, is the capital and one of the largest cities in the Middle East. The study included single or married female students without children, aged 18–38, who were pursuing master’s, doctoral, postdoctoral, Doctor of Medicine (MD), Master of Public Health (MPH), specialty, subspecialty, or fellowship degrees. Bachelor students were not included. The study focused on students aged ≤ 38, who are more likely to delay childbearing due to educational or career goals [27, 28].

From March to August 2022, students in Tehran universities were targeted through social media groups such as WhatsApp, Telegram, and Linked-in. Convenience sampling was used to invite interested students from all universities in Tehran to participate in the study. An online forum (https://survey.porsline.ir - professional account) was used to distribute the survey, which could be accessed through a hyperlink. Participants were not required to register or become members, and they could skip questions and review them all. However, it was not possible to submit incomplete questionnaires in order to reduce missing data. The survey advertisement and homepage included inclusion criteria for verification purposes. The homepage also provided a brief project description and OC procedure information for participants. Those who completed the survey were entered into a lottery to win one of 20 free 15 GB internet vouchers.

### Instrument

To evaluate beliefs and knowledge about OC for medical and social reasons, we utilized the Fertility Preservation Survey (FPS) instrument, developed by Daniluk and Koert in 2016 [18]. The questionnaire comprised of 7 demographic questions (Table 1), 9 questions about fertility intentions of students (Table 2), 9 questions about beliefs concerning the ideal timing of parenthood (5-point-scale, Table 3), 9 questions about beliefs about FP (4-point-scale, Table 4), 16 questions about decision-making considerations (4-point-scale, Table 5), and 12 questions about knowledge of egg freezing (5-point-scale, Table 6). This tool does not have a total score and each sub-scale was analyzed and reported separately, with higher scores indicating greater importance (Table 3), priority in decision making (Tables 4 and 5), and knowledge (Table 6). We also modified the classification of responses for knowledge of egg freezing by considering “definitely not” and “probably not” as a “NO” response, and “probably” and “definitely” as a “YES” response. An “uncertain” response indicated that the participant did not know the answer. A score of one was given for a correct answer, and a score of zero was given for an incorrect or “I do not know” response. The total score (range from 0 to 12) was obtained by adding the points of the 12 knowledge questions, with higher scores indicating greater knowledge of egg freezing. Furthermore, the participants provided their self-assessed current level of knowledge pertaining to FP, ranging from “no knowledge” to “some knowledge”, “fairly knowledgeable”, and “very knowledgeable”.

After obtaining permission from the developers of the tool, Daniluk and Koert (2016) [18], we translated the questionnaire from English to Persian using the forward-backward method. To ensure trans-cultural adaptation, 30 students and 5 experts in the fields of gynecology, embryology, midwifery, reproductive health, and sociology were invited to provide feedback on the questionnaire’s content and clarity before finalization. To investigate technical problems, the online questionnaire was pretested by 20 students who had similar characteristics with the participants of the main study. Subsequently, these questionnaires were excluded from the main study. The questionnaire’s reliability was assessed through Cronbach’s alpha coefficient, which indicated a score of ≥ 0.70 for both the entire scale and its domains.

### Sample size

The final sample size of the study was determined by selecting the largest value from the separately calculated sample sizes for each primary outcome (knowledge and belief about egg freezing in female students). To accurately estimate the knowledge about egg freezing in female students with a confidence level of 95%, a power of 80%, and a precise estimation of d = 0.05, a minimum sample size of 1245 people was required. This estimation was based on the estimated standard deviation of 0.9 in study of Daniluk and Koert (2016) [18].

### Data analysis

The data was transferred to SPSS (Chicago, Illinois, Version 16) from an Excel file for analysis. Descriptive statistical methods, such as mean (M) and standard deviation (SD), and analytical statistics, such as One-way ANOVA, were utilized to analyze the data. Any results with a p-value of less than 0.05 were considered statistically significant.

## Results

### Demographic characteristics

Out of 3903 visitors, 1279 eligible and interested students completed the online survey. The average age of participants was 26.38 ± 4.9 years. The majority of students were single (77.2%), of Fars ethnicity (54.8%), and pursuing a master’s degree (77.7%). About half of the participants had equal household income and expenses (52.4%), while 23.0% had lower income than expenses. Student paid employment (full-time or part-time) was prevalent in 34.8% of participants (Table 1).

**Table 1:** Demographic characteristics of students (N = 1279) and its relationship with fertility preservation knowledge

Demographic characteristics	Category	N (%)	Knowledge Score		Post Hoc Test
			**M** ± SD **	**p**	
Age; years*	< 25	539(42.1)	7.23 ± 2.22	^***^0.290	-
	25–29	435(34.0)	7.01 ± 2.20		
	30–34	193(15.1)	7.00 ± 2.01		
	35–38	112(8.8)	6.93 ± 2.01		
**Ethnicity**	Fars	701(54.8)	7.15 ± 2.20	^***^0.466	-
	Turks	239(18.7)	7.18 ± 2.27		
	Kurds	95(7.4)	6.96 ± 1.90		
	Lurs	83(6.5)	7.12 ± 1.76		
	Others	161(12.6)	6.83 ± 2.16		
**Marital status**	Single	989 (77.2)	7.04 ± 2.22	^***^ **0.028**	^1^a<^2^b
	Married	257 (20.3)	7.40 ± 1.98		
	Others (divorced, seprated, widowed)	33 (2.6)	6.64 ± 1.37		
**University course/faculty**	Medical sciences	355(27.8)	7.09 ± 1.96	^***^ **0.025**	^3^d<^4^a,^5^b,^6^c,^7^e
	Social sciences	275(21.5)	7.19 ± 2.23		
	Engineering	367(28.7)	7.18 ± 2.38		
	Art	62(4.8)	6.36 ± 2.22		
	Basic science	170(13.3)	7.25 ± 1.87		
	Others	50(3.9)	6.49 ± 2.21		
**Expected degree**	MSc	994 (77.7)	7.08 ± 2.21	^***^0.534	-
	PhD and post doc	246 (19.3)	7.21 ± 1.96		
	Medical residency	39 (3.1)	6.84 ± 2.04		
**Occupation**	Paid employment (Full time)	238(18.6)	6.88 ± 2.04	^***^ **0.048**	^8^a,^9^b<^10^d
	Paid employment (Part time)	207(16.2)	6.89 ± 1.95		
	Volunteer work	75(5.9)	6.95 ± 2.26		
	Unemployed	759(59.4)	7.24 ± 2.24		
**Income**	Household income less than expenses	294(23)	7.20 ± 2.16	^***^0.118	-
	Household income equal to expenses	670(52.4)	7.16 ± 2.22		
	Household income more than expenses	315(24.6)	6.88 ± 2.02		

* We selected these specific age categories arbitrarily based on our specific research questions, our criteria for including certain age groups, population demographics (such as age distribution in the samples), and to enable meaningful comparisons across various age groups.

**Score’s range: 0–12 ***One-way ANOVA.

^1^Single, ^2^ Married.

^3^ Art, ^4^ Medical sciences, ^5^ Social sciences, ^6^ Engineering, ^7^ Basic science.

^8^ Paid employment, ^9^ Paid employment, ^10^ Unemployed.

### Fertility intentions

The majority of participants expressed a desire to have two children in the future (40.5%), while a smaller percentage stated they did not want any children (16.3%). When it came to pregnancy and childbirth, most participants anticipated becoming pregnant with their first child between the ages of 30–34 (41.1%, M: 30.3 ± 4.13 years) and giving birth to their last child between the ages of 35–39 (46.7%, M: 35.28 ± 4.18 years). A significant percentage of students (62.7%) reported feeling disappointed, upset, and distraught if they were unable to bear children. Additionally, the majority of participants believed that the ideal age for a woman to have her first child was < 30 (52.3%), and that the oldest age a woman should consider bearing a child was ≥ 40 (64.5%) (Table 2).

**Table 2:** Fertility intentions among students (N = 1279)

Fertility intentions	Category	N (%)
**How many children do you hope to have?**	0	209(16.3)
	1	219(17.1)
	2	518(40.5)
	≥ 3	333(26.0)
**About how old do you expect to be when you become pregnant with your first child?**	< 30	420(39.3)
	30–34	440(41.1)
	35–39	185(17.3)
	> 40	25(2.3)
**If you intend to have more than one child, about how old do you expect to be when you give birth to your last child?**	< 30	61(5.7)
	30–34	326(30.5)
	35–39	500(46.7)
	> 40	183(17.1)
**About how many months do you expect it to take for you to get pregnant once you start trying?**	1–2	504(39.4)
	3–6	258(20.2)
	7–12	468(37.3)
	> 12	40(3.1)
**How would you feel if you were never able to bear a child?**	Not bothered	477(37.3)
	Disappointed	80(6.3)
	Upset	504(39.4)
	Distraught	218(17.0)
**What do you consider to be the ideal age for a woman to give birth to a child for the first time?**	< 20	8(0.6)
	20–24	149(11.6)
	25–29	669(52.3)
	30–34	362(28.3)
	35–39	64(5.0)
	≥ 40	27(2.1)
**What do you consider to be the oldest age a woman should consider bearing a child?**	< 30	15(1.2)
	30–34	69(5.4)
	35–39	437(34.2)
	40–44	441(34.5)
	45–49	230(18.0)
	≥ 50	87(6.8)

### Beliefs about the ideal timing of parenthood

According to Table 3, the top priorities for deciding when to become a mother were the items of “being with a partner who would be an involved and loving parent” (83.8%, M: 4.74 ± 0.69), “being in a stable relationship” (82.3%, M: 4.65 ± 0.87) and “being able to financially support a child” (81.5%, M: 4.71 ± 0.68).

**Table 3:** Beliefs about the ideal timing of parenthood in students (N = 1279)

Beliefs	Not Important N (%)	Little Important N (%)	Moderately important N (%)	Very important N (%)	Extremely important N (%)	M ± SD (5-point scale)*
Being in a stable relationship	40 (3.1)	18 (1.4)	53 (4.1)	115 (9.0)	1053 (82.3)	4.65 ± 0.87
Being able to financially support a child	11 (0.9)	13 (1.0)	63 (4.9)	150 (11.7)	1042 (81.5)	4.71 ± 0.68
Having completed my education	57 (4.5)	65 (5.1)	256 (20.0)	323 (25.3)	578 (45.2)	4.01 ± 1.12
Being established in my job/career	45 (3.5)	42 (3.3)	207 (16.2)	294 (23.0)	691 (54.0)	4.20 ± 1.05
Being able to stay at home to raise my child	138 (10.8)	208 (16.3)	418 (32.7)	249 (19.5)	266 (20.8)	3.23 ± 1.25
Being with a partner who would be an involved and loving parent	22 (1.7)	9 (0.7)	33 (2.6)	143 (11.2)	1072 (83.8)	4.74 ± 0.69
Being young enough and having the energy to be an involved parent	48 (3.8)	49 (3.8)	220 (17.2)	384 (30.0)	578 (45.2)	4.09 ± 1.05
Having a proper home in which to raise a child	18 (1.4)	31 (2.4)	129 (10.1)	301 (23.5)	800 (62.5)	4.43 ± 0.87
Being in good health	8 (0.6)	2 (0.2)	34 (2.7)	229 (17.9)	1006 (78.7)	4.73 ± 0.57

*5-point Likert scale ranges from 1(Not Important) to 5 (Extremely important).

### Beliefs about OC

Students “definitely” or “probably” agreed to preserve fertility with medical (93.3%) and social (86.9%) indications and endorsed that the costs of social (87.4%) and medical (95.1%) egg freezing should be covered by healthcare system. An overwhelming majority of students, with 95.5% expressing this belief, supported the inclusion of information about egg freezing as a routine part of healthcare visits for women of childbearing age by their physicians. When it comes to donating eggs, women have expressed that if they choose not to use their eggs to conceive a child themselves, they would “probably” or “definitely” consider donating them for fertility research (67.1%), to a friend or family member who is facing fertility issues (58.1%), or to couples who are struggling with infertility (42.7%) (Table 4).

**Table 4:** Beliefs about oocyte cryopreservation for medical and social reasons (N = 1279)

Beliefs about fertility preservation	Definitely not N (%)	Probably not N (%)	Probably N (%)	Definitely N (%)	M ± SD (4-point scale)*
If a woman isn’t ready to have a child in her 20’s or 30’s, she should consider preserving her fertility through egg freezing.	43 (3.4)	124 (9.7)	619 (48.4)	493 (38.5)	3.22 ± 0.75
If a woman wishes to freeze her eggs to preserve her fertility because she isn’t ready to have a child, the costs for this procedure should be covered by healthcare system.	41 (3.2)	120 (9.4)	450 (35.2)	668 (52.2)	3.36 ± 0.78
If a woman’s future fertility may be adversely affected due to cancer treatments, she should be able to freeze her eggs, to increase her chances of becoming a mother in the future.	26 (2.0)	59 (4.6)	403 (31.5)	791 (61.8)	3.53 ± 0.68
If a woman chooses to freeze her eggs as a way to preserve her fertility prior to undergoing cancer treatment, the costs for this procedure should be covered by healthcare system.	20 (1.6)	43 (3.4)	327 (25.6)	889 (69.5)	3.63 ± 0.62
As part of regular healthcare visits, physicians should routinely provide women of childbearing age with information about egg freezing.	15 (1.2)	42 (3.3)	407 (31.8)	815 (63.7)	3.58 ± 0.61
I would prefer to work for a company with a benefit package that includes the cost of egg freezing.	62 (4.8)	209 (16.3)	519 (40.6)	489 (38.2)	3.12 ± 0.85
If I couldn’t afford to freeze my eggs, I would consider accepting money from a parent or family member to pay for this procedure.	179 (14.0)	412 (32.2)	485 (37.9)	203 (15.9)	2.55 ± 0.91
I would consider freezing my eggs if I had not yet found a suitable partner with whom I could have children.	163 (15.2)	220 (20.6)	421 (39.3)	266 (24.9)	2.73 ± 0.99
I would consider freezing my eggs if I was not personally ready to have children.	120 (9.4)	249 (19.5)	575 (45.0)	335 (26.2)	2.87 ± 0.90
I would consider freezing my eggs if my partner was not ready to have children.	116 (9.1)	221 (17.3)	600 (46.9)	342 (26.7)	2.91 ± 0.89
I would consider freezing my eggs if I were facing cancer treatments that could harm my future fertility.	102 (8.0)	114 (8.9)	516 (40.3)	547 (42.8)	3.17 ± 0.89
If, after freezing my eggs, I decided not to use them to try to become pregnant, I would consider donating them for medical research.	178 (13.9)	231 (18.1)	436 (34.1)	434 (33.9)	2.88 ± 1.03
If, after freezing my eggs, I decided not to use them to try to become pregnant, I would consider donating them to a friend or family member with fertility problems, to help them conceive a child.	318 (24.9)	346 (27.1)	320 (25.0)	295 (23.1)	2.46 ± 1.09
If, after freezing my eggs, I decided not to use them to try to become pregnant, I would consider donating them to an anonymous egg donor program to help an infertile individual or couple conceive a child.	369(28.8)	358(27.9)	291(22.7)	261(20.0)	2.34 ± 1.1

*5-point Likert scale ranges from 1 (Definitely not) to 4 (Definitely).

### Decision-making considerations

The recommendation of the oncology team (92.6%, M: 3.46 ± 0.71), prognosis for full recovery (91.8%, M: 3.41 ± 0.81), and severity of cancer (89.1%, M: 3.46 ± 0.74) were the most important factors that “probably” or “definitely” affect the students’ decision-making regarding FP for medical reasons (Table 5).

**Table 5:** Factors that influence the decision making for fertility preservation for either social or medical reason in students (N = 1279)

Factors	Definitely not N (%)	Probably not N (%)	Probably N (%)	Definitely N (%)	M ± SD (4-point scale)*
**For social or medical reason**					
Cost of the procedure	107 (8.4)	205 (16.0)	415 (32.4)	552 (43.2)	3.10 ± 0.95
Current success rates and likelihood of achieving a viable pregnancy in the future using frozen eggs	48 (3.8)	166 (13.0)	537 (42.0)	528 (41.3)	3.20 ± 0.80
Number of times I would need to go through the procedure to harvest enough eggs	71 (5.6)	216 (16.9)	576 (45.0)	416 (32.5)	3.04 ± 0.84
Possible discomfort or side-effects from the hormone injections	40 (3.1)	110 (8.6)	464 (36.3)	665 (52.0)	3.37 ± 0.77
long-term risks to my health or future fertility from the hormones and egg retrieval process	41 (3.2)	84 (6.6)	424 (33.2)	730 (57.1)	3.44 ± 0.75
Possible health risks to a child conceived using frozen eggs	43 (3.4)	86 (6.7)	331 (25.9)	819 (64.0)	3.50 ± 0.76
Concerns about the negative judgments of others	681 (53.2)	279 (21.8)	168 (13.1)	151 (11.8)	1.83 ± 1.05
Ethical or moral concerns about fertility preservation in general	575 (45.0)	330 (25.8)	220 (17.2)	154 (12.0)	1.96 ± 1.05
Concerns about interfering with the “natural” fertility lifespan	401 (31.4)	269 (21.0)	366 (28.6)	243 (19.0)	2.35 ± 1.11
Concerns about having to decide what to do with any remaining frozen eggs once I completed my family or if I decided not to use my frozen eggs	317 (24.8)	374 (29.2)	365 (28.5)	223 (17.4)	2.38 ± 1.04
**If faced with cancer treatment**					
Whether or not I already had a child or children	102 (8.0)	144 (11.3)	520 (40.7)	513 (40.1)	3.12 ± 0.90
Severity of my cancer	62 (4.8)	78 (6.1)	400 (31.3)	739 (57.8)	3.41 ± 0.81
My prognosis for a full recovery	43 (3.4)	62 (4.8)	431 (33.7)	743 (58.1)	3.46 ± 0.74
Recommendation of my oncology team	39 (3.0)	55 (4.3)	458 (35.8)	727 (56.8)	3.46 ± 0.71
My partner’s feelings, if I was in a committed relationship	79 (6.2)	84 (6.6)	423 (33.1)	693 (54.2)	3.35 ± 0.85
How quickly the egg freezing procedure could be done/completed	49 (3.8)	141 (11.0)	587 (45.9)	502 (39.2)	3.20 ± 0.78
Concerns about the effects of the hormones or egg retrieval procedure on my health	39 (3.0)	112 (8.8)	523 (40.9)	605 (47.3)	3.32 ± 0.76
The importance of becoming a mother in the future	102 (8.0)	120 (9.4)	446 (34.9)	611 (47.8)	3.22 ± 0.91
Emotional support from my family and friends	85 (6.6)	154 (12.0)	478 (37.4)	562 (43.9)	3.18 ± 0.88

*4-point Likert scale ranges from 1 (Definitely not) to 4 (Definitely).

### Knowledge about FP

Participants described their current FP knowledge as “no knowledge” (12.3%), “some knowledge” (49.3%), “fair knowledgeable” (27.0%) and “very knowledgeable” (11.4%). It is worth noting that 93.7% of the respondents held the mistaken belief that a single round of treatment is typically enough to collect an adequate number of eggs for freezing. The majority of knowledge questions (9 out of 12) had an average score of approximately 3 (“uncertain”), with a standard deviation of around 1. This indicates that participants were unsure about their answers. (Table 5). When we utilized an alternative classification system (“yes,“ “no,“ and “don’t know”), it became apparent that there was a general uncertainty among participants regarding the correct answers to knowledge questions. In fact, it could be argued that almost 50% of respondents were unsure about the answer to each question (Table 6).

**Table 6:** Women’s knowledge about fertility preservation (N = 1279)

Knowledge Items		True/ False	Correct response N (%)	definitely not N (%)	probably not N (%)	Uncertain N (%)	Probably N (%)	Definitely N (%)	M ± SD (5-point scale)*
1	For women over 30, overall health and fitness level is a better indicator of fertility than age.	F	305 (32.2)	63 (4.9)	242 (18.9)	332 (26.0)	471 (36.8)	171 (13.4)	3.34 ± 1.08
2	Similar to IVF, egg freezing requires the injection of hormones to stimulate egg production and the surgical retrieval of eggs from a woman’s ovaries.	T	686 (89.8)	28 (2.2)	50 (3.9)	515 (40.3)	519 (40.6)	167 (13.1)	3.58 ± 0.84
3	The long-term health implications for children born using frozen eggs are currently unknown.	T	607 (80.0)	33 (2.6)	119 (9.3)	520 (40.7)	468 (36.6)	139 (10.9)	3.43 ± 0.89
4	One cycle of treatment is usually sufficient to retrieve enough eggs for freezing.	F	58 (6.3)	19 (1.5)	39 (3.0)	352 (27.5)	655 (51.2)	214 (16.7)	3.78 ± 0.80
5	In Iran the cost of one cycle of egg freezing is about $100 to $200 USD, including medications.	F	297 (38.2)	81 (6.3)	216 (9.0)	502 (39.2)	375 (29.3)	105 (8.2)	3.16 ± 1.00
6	There is a significant decrease in a woman’s ability to become pregnant after the age of 37.	T	989 (91.9)	26 (2.0)	61 (4.8)	203 (15.9)	619 (48.4)	370 (28.9)	3.97 ± 0.90
7	The egg freezing procedure poses significant risks to a woman’s health and future fertility.	T	275 (41.1)	96 (7.5)	298 (23.3)	610 (47.7)	202 (15.8)	73 (5.7)	2.88 ± 0.95
8	The age of the woman at the time she elects to use her frozen eggs, is more significant in achieving a viable pregnancy, than the age of the woman at the time her eggs were frozen.	F	496 (56.5)	151 (11.8)	345 (27.0)	401 (31.4)	283 (22.1)	99 (7.7)	2.87 ± 1.12
9	Egg freezing before the age of 35 can significantly prolong a woman’s fertility.	T	785 (85.1)	28 (2.2)	109 (8.5)	357 (27.9)	607 (47.5)	178 (13.9)	3.62 ± 0.90
10	A woman can successful and safely use eggs frozen when she was still fertile, to become pregnant in her 40’s and 50’s.	T	565 (77.2)	31 (2.4)	136 (10.6)	547 (42.8)	441 (34.5)	124 (9.7)	3.38 ± 0.88
11	There are no side effects associated with the hormone injections required for egg freezing.	F	623 (81.1)	188 (14.7)	435 (34.0)	511 (40.0)	100 (7.8)	45 (3.5)	2.51 ± 0.95
12	Most frozen eggs will survive the defrost process and be able to be fertilized.	F	250 (40.7)	43 (3.4)	207 (16.2)	665 (52.0)	307 (24.0)	57 (4.5)	3.10 ± 0.83

*5-point Likert scale ranges from 1(Definitely not) to 5 (Definitely).

Table 1 reveals that, among the demographic variables analyzed, the marital status (P = 0.028), university faculty (P = 0.025) and occupation (P = 0.048) were significantly associated with the knowledge of FP. According to Tukey’s post hoc test, it was found that the FP knowledge among married students (M: 7.40 ± 1.98) was significantly higher than single counterparts (P = 0.045). However, no significant differences were observed in any other comparisons. Tukey’s post hoc test revealed that the level of FP knowledge among students in the Art faculty (M: 6.36 ± 2.22) was significantly lower compared to students in the Medical Sciences, Social Sciences, Engineering, and Basic Science faculties (all P < 0.05). However, there were no significant differences found in other comparisons. Tukey’s post hoc test revealed that unemployed students had a significantly higher level of FP knowledge (M: 7.24 ± 2.24) compared to both full-time employed students (P = 0.04825) and part-time employed students (P = 0.041). However, no significant differences were observed in the remaining comparisons.

## Discussion

As far as we know, this is the first research to evaluate female students’ knowledge and beliefs regarding OC for social and medical reasons in Tehran, Iran, and the factors that may influence their decision-making. The results indicate that a significant number of students plan to have their first and last child during a period when female fertility is declining, which is in line with Meissner et al.‘s research [11]. Postponing childbearing also is a growing trend in Iran [22]. Despite positive attitudes about childbearing, a wide range of sociocultural and economic factors encourage women to defer the first pregnancy [29]. Health care providers can explain about the importance of childbearing during their most fertile years, age-related decline, and the potential regret of delaying childbearing in order to increase fertility awareness [30]. According to a study in Iran, the reproductive-age population has limited knowledge about fertility. The study recommends including fertility awareness education in health systems, high school/university courses, and community educational campaigns [31].

Despite the significant drop in Iran’s fertility rate, a surprising 66.5% of students plan to have at least two children in the future and value fertility. In fact, 62.7% of participants would feel disappointed, upset and distraught if they were unable to have children, which may be due to the continued importance of fertility and childbearing in Iranian culture [32]. However, because of socio-economic constraints in Iran, the family size has been restricted and consequently, women are unable to fulfill their fertility aspirations and ultimately give birth to fewer children than they intended, resulting in a fertility rate below replacement level [33].

The decision to have a child was heavily influenced by having a partner who is an involved and loving parent, having stable relationship and being able to financially support a child. Recent studies in Iran have also highlighted the significance of marital satisfaction, positive partnership quality, and social support in the decision-making process for childbearing [34, 35].

Consistent with previous research [18, 19, 26, 36], a high percentage of students (> 80%) agreed to preserving fertility with medical and social indications and endorsed that the costs of medical and social egg freezing should be covered by healthcare system. However, these results can only be generalized to students, because they are more intended to postpone the childbearing due to pursuing higher levels of education or career development. Additionally, expressing favorable views towards FP without comprehending its benefits and drawbacks could pose challenges. To make informed decisions about oocyte cryopreservation, it’s crucial to be aware of its efficacy, cost-effectiveness, and ethical considerations. Cost was a significant factor for 75.6% of participants who chose to preserve their fertility. However, only 15.9% of students were willing to accept money from family members to pay for the procedure, and 65.3% were unemployed. Therefore, insurance coverage for this service is crucial, especially for students. Iran’s ‘Youthful Population and Protection of the Family’ law [37] was recently approved to promote childbearing and prioritize the detection of infertility risks (Article 42). However, FP has been overlooked in this supportive policy and is not currently covered by insurance system.

Participants were more willing to donate their unused eggs for research than for infertility treatments of their family/friends and other infertile couples. Consistently, the majority of female university students in Italy were less likely to donate their unused eggs for infertility treatments in family/friends and other infertile couples compared to research purposes. Specifically, 64.9% were not in favor of donating to a known woman or couple, while 57.5% were not willing to donate to a biobank [38]. Around 90% of participants in a UK study expressed willingness to donate oocytes for research or to infertile women if they never need them [39], but in Iran, there is less enthusiasm for third-party reproduction due to insufficient knowledge and sociocultural, religious, and legal limitations. Iranian couples are cautious about non-biological parenting and make decisions step-by-step, likely due to the importance of lineage and biological relatedness in Islam [40].

The oncology team’s advice and the likelihood of full recovery were crucial factors for participants in choosing to preserve their eggs in the event of cancer. However, it has been demonstrated that Iranian oncologists require further education on FP options [25]. When a woman receives a cancer diagnosis, particularly gynecological cancer, it is crucial for the fertility specialists and oncology team to collaborate and provide well-informed counseling regarding potential future infertility and personalized FP options [41]. Furthermore, surgery, chemotherapy, and radiation can negatively impact physical, sexual, and emotional well-being, highlighting the need for suitable fertility-related psychological support. Additional research is needed to assess the impact of multidisciplinary FP counseling on cancer patients’ quality of life [42, 43].

Gaps in knowledge about FP and need to detailed information about FP was reported among students. The correct response rate to knowledge questions was 57.7% which was relatively moderate and 61.6% of the students evaluated themselves as having no or little FP knowledge. Two studies conducted in China found that students had a high level of recognition regarding social OC but lacked knowledge about it [19, 44]. The overall correct response rate of childless Canadian women to FP questions was 33% [18] which may be associated with the differences in the study groups (childless women compared to childless students). Experts recommend that women who desire parenthood and are within the optimal age range for FP receive accurate information about it and actively participate in decision-making regarding the procedure [45]. In the present study, nearly all participants (95.5%) believed that it is necessary to routinely provide information about egg freezing to women of childbearing age as part of regular healthcare visits with health professionals. To promote informed decision-making about fertility, it is essential to provide comprehensive fertility education to all women while respecting their individual autonomy [46].

Students who were married and unemployed showed higher levels of knowledge regarding FP, while those studying in the Art faculty had lower levels of knowledge compared to students in other fields. In a comparable study conducted in Canada, there was no correlation between age or income and self-rated knowledge. However, single women in the study rated themselves as less knowledgeable compared to women with partners [18]. In Iran’s public health system, the emphasis is on maternal and child health, resulting in unmarried and childless women receiving fewer services. This issue should be addressed in future policies concerning reproductive health.

The study’s results provide valuable insights into female students’ knowledge and beliefs about OC in an Islamic country in the Middle East and Asia. The study’s strengths include its inclusion of students from various academic programs, both medical and non-medical, who are typically from different regions across Iran and studying at universities in Tehran. Conducting the survey online was a decision made due to limited access to all universities in Tehran and the occurrence of a new wave of Covid-19 at the time of the study. There are some methodological limitations that are commonly associated with online surveys. The collection of informed consent through an online application without any doctor-patient counseling or signature raises concerns about the validity of the informed consent. However, researchers provided enough information about the study so that participants could make an informed decision about whether to participate. We also assured participants that their personal information will be kept confidential and that their data will be used only for the purposes of the study. Despite having information on the number of visits and completion rate (32.7%) in this survey, it is difficult to accurately estimate the response rate. The reliability of the questionnaire’s visits is questionable due to potential ineligible participants opening the link out of curiosity or multiple openings by one person. Nonresponse bias is a concern as those not interested in childbearing may have ignored the research advertisement while those interested in preserving fertility may have noticed it more. For this reason, researchers offered incentive to encourage participation. However, participants could also be more likely to start and complete the survey due to the lottery incentive. As such, it is important to interpret the findings with caution due to these limitations, and it is also crucial to recognize that cross-sectional studies have predictive limitations and cannot establish causality. Despite all these limitations, the online platform allowed for a large sample size, which increased the study’s statistical power and generalizability of findings.

Future studies should investigate women’s experiences with egg freezing, as well as its success rates and potential risks. There is a lack of research on the psychological effects of FP counseling and treatments [43]. Future intervention studies should determine if accurate and sufficient information about indications, efficacy, and cost-effectiveness affects students’ inclination towards practicing FP. While the current study focused on female students’ knowledge and beliefs about OC, future studies should target both men and women.

## Conclusions

The research conducted revealed that female students from Tehran universities had a positive attitude towards medical and social egg freezing, but lacked the necessary knowledge of the ideal timing for childbearing. The majority of students expressed a desire to have at least two children in the future, indicating that fertility was important to them; however, they were not aware of the impact age has on fertility. To make informed decisions regarding FP, it is important for students to have detailed information about the costs, risks, and success rates of the procedure, as well as the limited number of oocytes that can be retrieved during each cycle and the fact that FP does not guarantee pregnancy. This information could be provided by healthcare professionals or [18] as part of a post-graduate course curriculum in universities. Improving knowledge and understanding of the benefits and drawbacks of FP can aid individuals in making informed decisions. Despite the fact that FP is currently not covered by Iran’s insurance system and remains largely unaffordable due to its high costs, recent policies that promote childbearing in Iran should be expanded to include FP for both medical and social reasons in order to address delayed childbearing and unintended childlessness. These findings are useful in developing FP services and education campaigns aimed at increasing awareness and knowledge of FP among students in Iran. Additionally, these results could inform government policies and public education strategies that seek to support childbearing among young women who may postpone their first birth due to not finding a partner until their thirties or are married but wish to prioritize their careers or education.

## Data Availability

The datasets used and analyzed in the current study, as well as the Persian version of the questionnaire can be provided by the corresponding author upon a reasonable request.

## List of abbreviations

FP: Fertility Preservation
OC: Oocyte Cryopreservation
